# Detection of microplastics stress on rice seedling by visible/near-infrared hyperspectral imaging and synchrotron radiation Fourier transform infrared microspectroscopy

**DOI:** 10.3389/fpls.2025.1645490

**Published:** 2025-07-21

**Authors:** Chaojie Wei, Hongxin Xie, Wei Wang, Yu-Feng Li, Xiaorong Wang, Ziwei Song, Fajun Chen

**Affiliations:** ^1^ College of Engineering, China Agricultural University, Beijing, China; ^2^ Chinese Academy of Sciences - The University of Hong Kong (CAS-HKU) Joint Laboratory of Metallomics on Health and Environment, Institute of High Energy Physics, Chinese Academy of Sciences, Beijing, China; ^3^ CAS Key Laboratory for Biomedical Effects of Nanomaterials and Nanosafety, Beijing Metallomics Facility, Institute of High Energy Physics, Chinese Academy of Sciences, Beijing, China; ^4^ National Consortium for Excellence in Metallomics, Institute of High Energy Physics, Chinese Academy of Sciences, Beijing, China

**Keywords:** microplastics, rice seedlings, visible/near-infrared hyperspectral imaging, synchrotron radiation-based Fourier transform infrared spectroscopy, deep learning

## Abstract

**Introduction:**

Microplastics (MPs), as emerging environmental contaminants, pose a significant threat to global food security. In order to rapidly screen and diagnosis rice seedling under MPs stress at an early stage, it is essential to develop efficient and non-destructive detection methods.

**Methods:**

In this study, rice seedlings exposed to different concentrations (0, 10, and 100 mg/L) of polyethylene terephthalate (PET), polystyrene (PS), and polyvinyl chloride (PVC) MPs stress were constructed. Two complementary spectroscopic techniques, visible/near-infrared hyperspectral imaging (VNIR-HSI) and synchrotron radiation-based Fourier Transform Infrared spectroscopy (SR-FTIR), were employed to capture the biochemical changes of leaf organic molecules.

**Results:**

The spectral information of rice seedlings under MPs stress was obtained by using VNIR-HSI, and the low-dimensional clustering distribution analysis of the original spectra was conducted. An improved SE-LSTM full-spectral detection model was proposed, and the detection accuracy rate was greater than 93.88%. Characteristic wavelengths were extracted to build a simplified detection model, and the SHapley Additive exPlanations (SHAP) framework was applied to interpret the model by identifying the bands associated with chlorophyll, carotenoids, water content, and cellulose. Meanwhile, SR-FTIR spectroscopy was used to investigate compositional changes in both leaf lamina and veins, and two-dimensional correlation spectroscopy (2DCOS) was employed to reveal the sequential interactions among molecular components.

**Discussion:**

In conclusion, the combination of spectral technology and deep learning to capture the physiological and biochemical reactions of leaves could provide a rapid and interpretable method for detecting rice seedlings under MPs stress. This method could provide a solution for the early detection of external stress on other crops.

## 1 Introduction

Microplastics (MPs), as emerging environmental pollutants, have been continuously threatening the ecosystem along with the growth of the global plastic industry (Thompson et al., 2024). Studies have demonstrated that less than 10% of the approximately 360 million tons of plastics produced globally were recycled, with the remainder being gradually decomposed into MPs particles through photo-oxidation, mechanical abrasion, and biodegradation to enter natural environment (Lwanga et al., 2022). Rice, a staple crop that supports nearly 60% of the global population, is vulnerable to MPs contamination (Kedzierski et al., 2023). Pathways such as polluted irrigation water, residual and fragmented agricultural plastic films, and atmospheric deposition could introduce MPs into rice paddies, thereby directly impairing crop growth and physiological functions (Kedzierski et al., 2023). MPs have been shown to physically block intercellular spaces in root epidermal cells, impeding water uptake, while also triggering reactive oxygen species (ROS) bursts and damaging chloroplast ultrastructure (Khan et al., 2024). These physiological disruptions lead to decreased chlorophyll content, reduced activity of key enzymes in the photosynthetic electron transport chain, and diminished plant biomass during the seedling stage (Yang et al., 2021). Additionally, the heavy metals and persistent organic pollutants adsorbed on their surfaces pose serious health risks by inducing metabolic disorders and organ damage through biomagnification (Lwanga et al., 2022). More notably, MPs can migrate and accumulate along the “soil-rice-human body” chain, having already been detected in human blood, lungs, and placentas (Tang et al., 2024). Therefore, establishment of early-stage MPs stress detection method in rice seedlings is not only fundamental for ensuring food production safety but also crucial in preventing pollutant transmission through the food chain, with far-reaching implications for both food security and public health.

Currently, widely used detection methods, such as microscopy (Carr et al., 2016), spectroscopy (Xu et al., 2019), mass spectrometry (Lee and Chae, 2021) and other emerging technologies, have demonstrated high recognition capabilities and are capable of *in-situ* detection. However, these methods typically require destructive sampling, complex pretreatment procedures, and specialized technical expertise, rendering them unsuitable for non-destructive and rapid detection in plants exposed to MPs. As an alternative, the physiological and biochemical responses of plant leaves under MPs stress offer a promising avenue for indirect detection (Yadav et al., 2022). For instance, exposure of cucumber plants to polystyrene (PS) MPs has reduced the contents of chlorophyll a/b, carotene and soluble sugar (Li et al., 2020). Similarly, spinach exposure to MPs leads to a decline in protein levels and abnormal accumulation of nitrites (Wang et al., 2024). upon MPs exposure, other crops such as maize (Sun et al., 2021), Chinese cabbage (Yang et al., 2021), tomatoes (Hernández-Arenas et al., 2021), and wheat (Lian et al., 2020) also exhibit marked biochemical alterations in leaf tissues, affecting pigments, enzyme activities, and biomass. Hyperspectral imaging (HSI) technology, by combining the spectral signatures of molecular vibrations with spatial imaging, provides a non-invasive method to assess biochemical changes (Lu et al., 2022). By capturing absorption features related to fundamental, harmonic, and combination vibrations of functional groups such as C–H, N–H, and O–H, HSI enables detailed exploration of alterations in plant organic constituents induced by MPs stress. Specifically, MPs are known to degrade photosynthetic pigments, producing characteristic absorption peaks for chlorophyll a at 680 nm (Zolotukhina et al., 2024) and for carotenoids at 470–500 nm (Sytar et al., 2017). Moreover, the stress-induced synthesis of phenolic compounds, such as ferulic acid, could result in broad fluorescence emission bands within the 640–660 nm range (Tran et al., 2022). Compared with traditional destructive techniques, HSI acquires molecular vibration data corresponding to the physiological responses of leaves, providing a feasible and effective tool for the monitoring of rice seedlings under MPs stress.

To achieve accurate detection and enhance the interpretability of MPs stress in plants, this study integrates machine learning and deep learning to extract and analyze key characteristics from hyperspectral image. Given the presence of noise, baseline drift and scattering interference in original spectra, appropriate preprocessing techniques are employed to eliminate these artifacts and improve data reliability (Bonah et al., 2019). To address the redundancy and complexity inherent in high-dimensional spectral data, principal component analysis (PCA) is utilized for dimensionality reduction and low-dimensional feature visualization (Marukatat, 2023). Furthermore, the spectral data is processed as sequence information to explore the intrinsic correlations. In this context, an attention-based long short-term memory (LSTM) network is constructed to address LSTM’s limitation in prioritizing critical spectral bands across long sequences, building a detection model that dynamically allocates weights to spectral features and thereby captures relevant sequence dependencies (Smagulova and James, 2019). However, a major challenge in applying deep learning lies in its black-box nature, which limits the model’s interpretability. To overcome this limitation, the SHapley Additive exPlanations (SHAP) framework is introduced to decode the model’s decision-making process by quantifying the contributions of individual wavelengths to the prediction outcome (Salih et al., 2025). This approach provides both predictive accuracy and explanatory transparency in assessing MPs induced stress in rice seedling.

Fourier transform infrared (FTIR) spectroscopy, as a complementary technique to the macroscopic VNIR-HSI, chemically characterizes the functional groups in the infrared region at the microscopic scale (Bondu et al., 2024). FTIR has advantages in studying plant microstructures and tissue levels responses under environmental stress. For instance, FTIR spectroscopy has been employed to monitor changes in lipids, proteins, and carbohydrates in corn leaves, thereby elucidating the relationship between photosynthetic activity and the concentration of active biochemical agents in response to external stimuli (Tran et al., 2025). Similarly, in Arabidopsis eceriferum subjected to drought stress, alterations in cuticular structure were characterized by analyzing spectral peak areas associated with CH stretching, asymmetric and symmetric CH_2_ modes, ester carbonyl groups, and asymmetric vibrations of C=O and CH_2_, highlighting the plant’s physiological adaptation mechanisms (Liu et al., 2020). These findings collectively confirm the feasibility of FTIR to investigate stress responses in rice seedlings exposed to MPs. With the advancement of synchrotron radiation (SR) technology, synchrotron-based FTIR (SR-FTIR) microspectroscopy has significantly enhanced spatial resolution and brightness, while maintaining a high signal-to-noise ratio (Du et al., 2021). This technique allows for fine-scale mapping of functional groups and compound distributions within microstructures such as mesophyll tissues and leaf veins, enabling simultaneous structural analysis, spatial localization of chemical components, and real-time monitoring of subtle compositional changes. However, distinguishing fine and dynamic spectral variations, such as sequential changes in rice leaf components under microplastic-induced stress, remains a significant challenge. Two-dimensional correlation spectroscopy (2DCOS) enhances spectral resolution and temporal sequencing of molecular responses, and has already proven effective in studies involving plant microstructural interactions and dynamic stress analysis (Park et al., 2015).

This study aims to develop a rapid detection method for assessing MPs stress in rice seedling and to explore the associated micro-scale interaction mechanisms. At the macro level, VNIR-HSI was used to analyze spectral differences in rice leaves under MPs exposure, and high-dimensional spectra were visualized through dimensionality reduction techniques. An SE-LSTM model was constructed to rapidly detect stress concentrations, and characteristic wavelengths were identified to build simplified models. The importance of each spectral band was further investigated using model interpretation methods. At the microscopic level, SR-FTIR combined with 2DCOS was employed to investigate the biochemical interactions between leaf veins and mesophyll tissues. To the best of our knowledge, this is the first report to assess MPs induced plant toxicity via spectral sensing of physiological and biochemical responses in leaves. The proposed approach may also be extended to assess the toxicity of other emerging environmental pollutants.

## 2 Materials and methods

### 2.1 Samples preparation

MPs were purchased from HengfaSuhua (Guangdong, China). The MPs size was measured using a dynamic light scattering (DLS) particle size analyzer (Zetasizer Nano ZS90, UK), with PET, PS and PVC exhibiting primary size distributions of 4.7 ± 3.1 μm , 6.5 ± 3.5 μm and 5.2 ± 2.7 μm , respectively. The cultivation of rice (Liangyou Y900) followed the method described by Xie et al (Xie et al., 2024). Fifty rice seeds were placed in each glass petri dish, and 5 mL of 10 or 100 mg/L PET, PS, and PVC MPs suspensions were added to the dishes, with six replicates for each treatment. Rice cultivated with sterile water was used as the control group. Under dark conditions at a temperature of 30°C and a relative humidity of approximately 60%, 5 mL of suspension or sterile water was added to each petri dish daily for seven days to promote germination. After the germination phase, the addition of MPs suspensions was stopped, and the petri dishes were transferred to alternating light and dark conditions for continued cultivation 35 days. The cultivation environment included a relative humidity of 60%, a daytime temperature of 28°C with a light intensity of 300–350 μmol·m^−2^·s^−1^ for 14 hours, followed by a nighttime temperature of 20°C in complete darkness for 10 hours. Hoagland solution was added periodically to ensure adequate nutrients for rice growth. One or two leaves were collected from each rice seedling, amounting to 300 leaves per treatment group and 2100 leaves across all groups.

### 2.2 VNIR-HSI acquisition

The VNIR-HSI of leaves of rice seedling under MPs stress were acquired by self-built push-broom line-scan hyper-spectrometer system, which consisted of several components: a spectrometer (ImSpector V10E), a CCD detector (Bobcat2.0), a variable focal-length lens (Schneider Xenoplen), a motorized translation stage, two 500 W tungsten-halogen lamps, and a computer for data acquisition. More detailed information about the system was provided in the previous research (Jiang et al., 2019). To obtain the VNIR-HSI spectra of entire leaves, the samples were placed on a white Teflon board measuring 15 × 30 cm, which was positioned on a translation stage. Once the board entered the camera’s field of view, hyperspectral images were captured with line scanning. At the same time, to prevent leaves damage caused by prolonged exposure to the high temperatures of halogen lamps, each sample was scanned and completed within 20 s. The hyperspectral images contained 300 spectral bands ranging from 380 to 1013 nm, with a spectral resolution of 2.2 nm. To reduce noise interference at the spectral boundaries, 256 wavebands within the range of 425 to 965 nm were retained.

**Figure 1:**
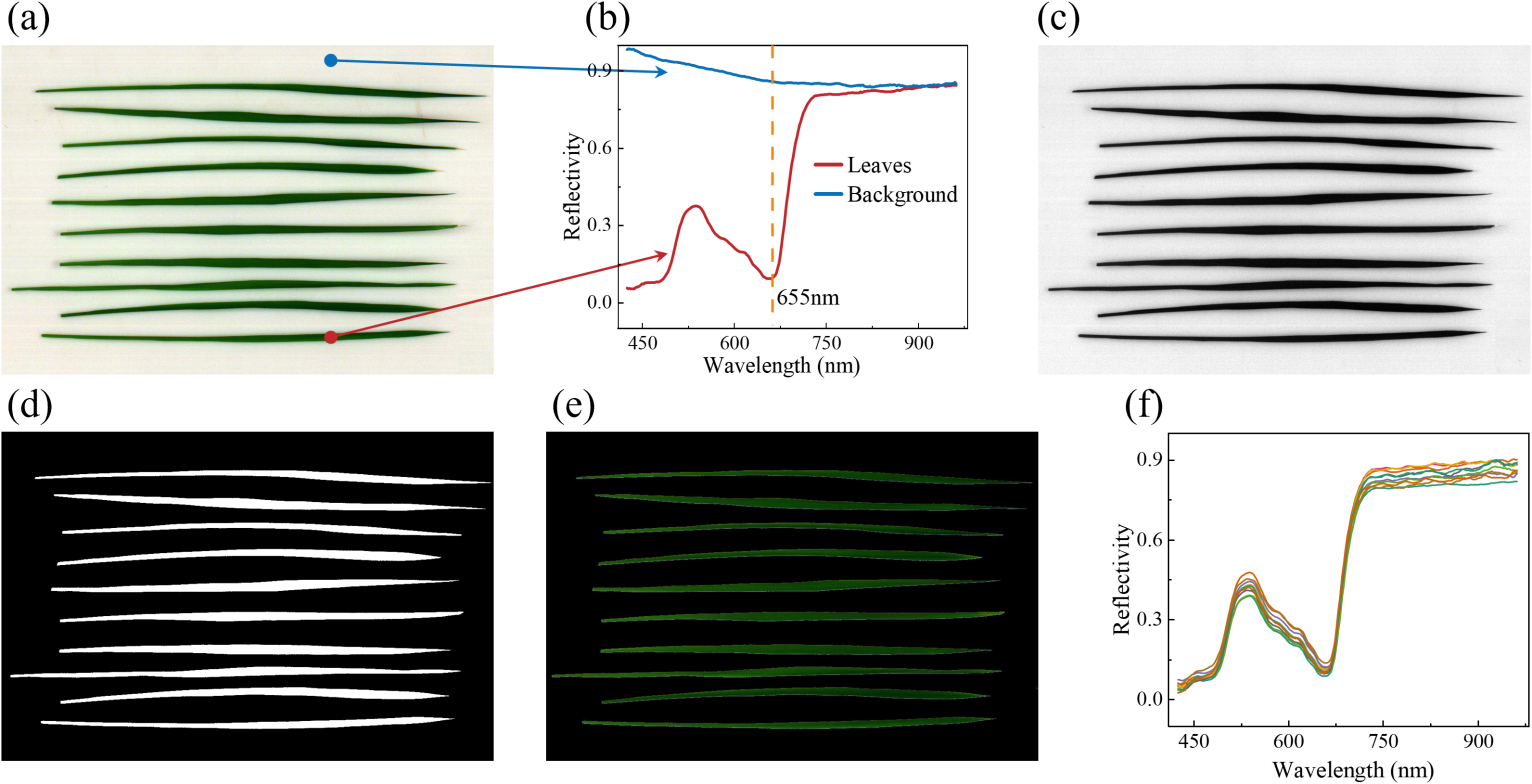
The extraction process of macro VNIR spectra. **(a)** Original pseudo-color image; **(b)** spectra of the background and leaves; **(c)** Grayscale images at 655nm; **(d)** Mask image; **(e)** Apply the original image to the mask image to remove the background; and **(f)** Mean spectra of each leaf.

Due to the narrow and elongated shape of the rice leaves, ten leaves were placed on the Teflon board simultaneously during sample collection, resulting in the pseudo-color image shown in **Figure 1a**. The reflectance spectra (**Figure 1b**) of the background and the leaves exhibited significant differences, particularly at 655 nm. Based on above features, the gray image (**Figure 1c**) at 655 nm was selected for background removal. A fixed threshold of 0.6 was applied to distinguish the leaves from the background, producing a mask image (**Figure 1d**) that clearly outlined the leaves contours without noise. The mask image was then overlaid onto the original image to remove the background, as shown in **Figure 1e**. Finally, the average spectral profile of each leaf was extracted (**Figure 1f**), and this process was repeated for all leaves to ensure comprehensive spectral data extraction.

### 2.3 SR-FTIR microspectroscopy and 2DCOS analysis

In order to further investigate the potential microscopic interaction mechanisms of the composition and structure of rice seedlings exposed to MPs, the SR-FTIR measurement was performed at a BL01B beamline at the National Synchrotron Radiation Laboratory (NSRL, China). The SR-FTIR photon source is equipped with a Bruker Vertex 70v Fourier transformation spectrometer and Bruker Hyperion 3000 microscope. After being embedded and fixed with Optimal Cutting Temperature (OCT) compound, the leaves were sectioned into 10 um thick slices using a cryo-microtome (Leica CM1850, Nussloch, Germany) and then placed on a BaF_2_ substrate for infrared microscopic imaging. The spectral imaging was obtained by raster scanning across the regions of interest, 20 × 20 μm^2^ for leaf vein and mesophyll regions of rice leaves. The SR-FTIR data were acquired in a transmission mode from 4000 to 800 cm^−1^ at a resolution of 4 cm^−1^ spectral with 32 scans. All of the SR-FTIR spectra were acquired using Bruker OPUS 8.5 software. A background spectrum for each image was acquired on a portion of the pure BaF_2_ slide. To obtain high-quality spectra, the SR-FTIR spectra were subtracted from the background spectrum. The transmission spectra were then converted into reflection spectra based on the Lambert-Beer law, followed by baseline correction to remove baseline drift from the reflection spectra.

Two-dimensional correlation spectroscopy (2DCOS) extends one-dimensional spectra to two-dimensional planes and provides the relationships between the absorption peaks of various molecular functional groups in complex samples through synchronous and asynchronous spectra (Park et al., 2015). Not only can the source of substances be confirmed, but also the sequence of vibration changes of each functional group can be clarified. Critically for fluorescent components, 2DCOS resolves emission sequence dynamics under perturbations: the signs (+/-) of asynchronous cross-peaks between distinct fluorophore bands determine the sequential order of quenching events and conformational rearrangements in light-responsive molecular systems. In the synchronous spectra, the intensity of the autocorrelation peak located on the main diagonal reflects the response to interference. The asynchronous spectral cross peaks are antisymmetric along the main diagonal and are composed only of the cross peaks on both sides of the diagonal. The cross peaks can be positive or negative, and the symbols of the cross peaks in the asynchronous spectrum diagram can be used to assist in determining the change order of the spectral bands during the external disturbance process.

### 2.4 Model development, evaluation, and explanation

The Squeeze-and-Excitation (SE) Block enhances the representational capacity of neural networks by exploring inter-channel dependencies. Through global contextual learning, it dynamically emphasizes characteristic wavelengths and suppresses unreliable ones. On the other hand, Long Short-Term Memory (LSTM), a type of recurrent neural network (RNN), excel at capturing sequential data and establish dependencies, effectively addressing issues like vanishing and exploding gradients in long time-series data. In this study, spectral data is simulated as time-series input to explore the relationships between spectral wavelengths. By combining the strengths of SE Block and LSTM, the SE-LSTM model was proposed, which enhanced features extraction, improved the detection and identification of MPs stress in rice, and provided a powerful method for processing complex spectral information.

**Figure 2:**
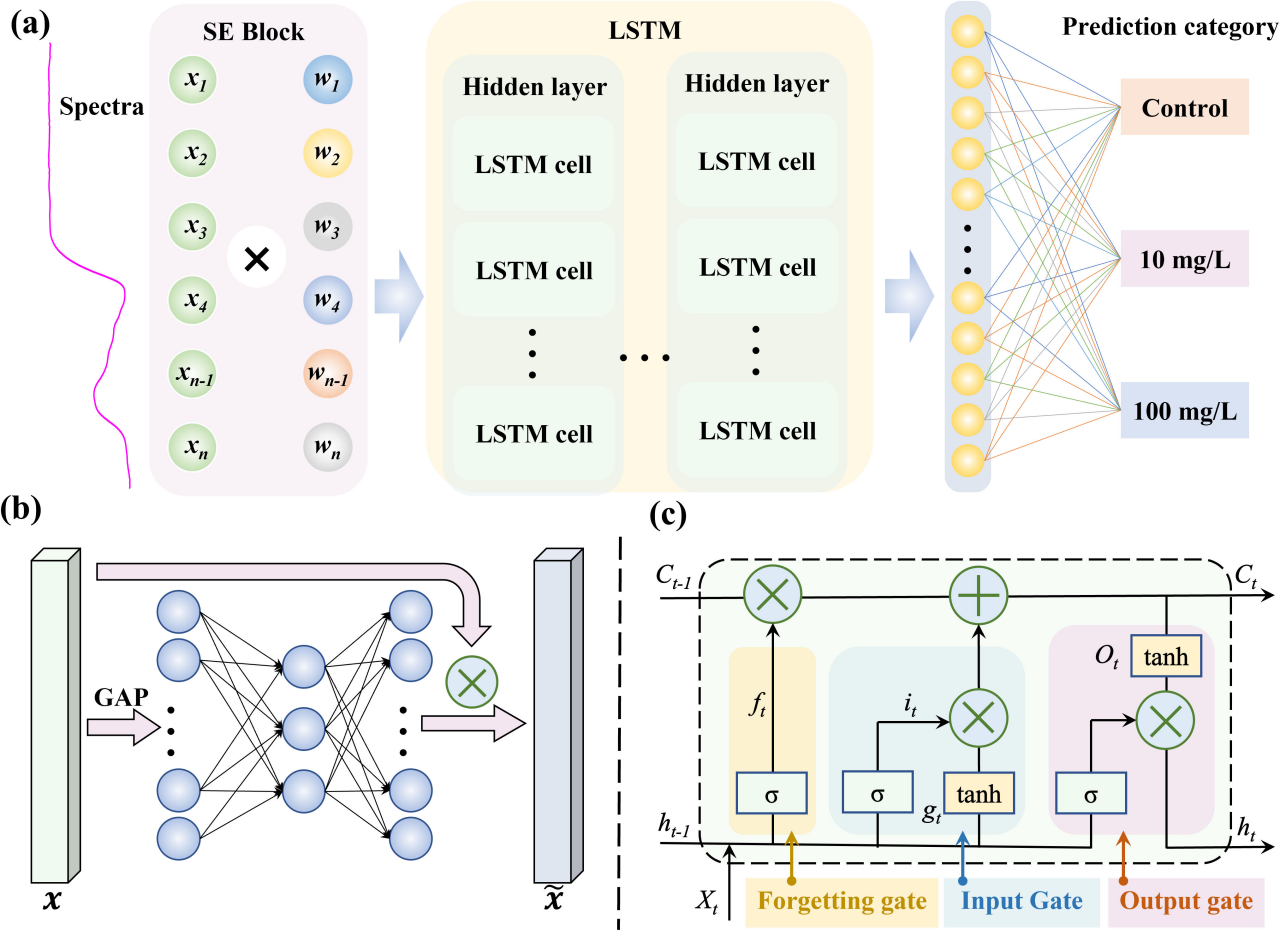
The framework of SE-LSTM model. **(a)** Overall structure of the model; **(b)** SE block attention mechanism structure; **(c)** LSTM cell structure.


The SE-LSTM model framework (**Figure 2a**) consists of three main components: the SE-Block, LSTM, and fully connected classification layers. Initially, the spectral data of length 256 is reshaped into a vector with 256 channels, followed by channel attention extraction using the SE Block (**Figure 2b**). Global average pooling (GAP) is applied to by the SE Block to capture global information, and then the features are excited through a lightweight fully connected network to reweight the channel-wise features. The LSTM network is composed of multiple sequential layers of LSTM cells (**Figure 2c**), each containing three main gating mechanisms: the input gate ( $i_{t}$ ), forget gate ( $f_{t}$ ), and output gate ( $o_{t}$ ). The forget gate determines which information from the previous cell state ( $C_{t-1}$ ) is retained or discarded based on the current input spectra ( $X_{t}$ ) and previous output ( $h_{t-1}$ ), while the input gate controls how much of the current input ( $g_{t}$ ) is written into the cell state. The output gate determines which information is output from the cell state, generating the new cell state ( $C_{t}$ ), which is used to produce the new output ( $h_{t}$ ) for the next layer or prediction. By iterating through these cells, the LSTM effectively captures long-term dependencies in sequential data, enabling the model to selectively remember or forget information. Finally, a fully connected layer is used to predict the microplastic stress concentration. The detailed calculation of LSTM as shown in Equations 1–6.

(1) $$f_{t}=\sigma \left(W_{fx}X_{t}+W_{fh}h_{t-1}+b_{f}\right)$$

(2) $$i_{t}=\sigma \left(W_{ix}X_{t}+W_{ih}h_{t-1}+b_{i}\right)$$

(3) $$o_{t}=\sigma \left(W_{ox}X_{t}+W_{oh}h_{t-1}+b_{o}\right)$$

(4) $$g_{t}=\text{tanh}\left(W_{gx}X_{t}+W_{gh}h_{t-1}+b_{g}\right)$$

(5) $$c_{t}=f_{t}\times c_{t-1}+i_{t}\times g_{t}$$

(6) $$h_{t}=o_{t}\times \text{tanh}\left(c_{t}\right)$$

Where $W_{fx}$ , $W_{ix}$ , $W_{ox}$ , $W_{gx}$ and $W_{fh}$ , $W_{ih}$ , $W_{oh}$ , $W_{gh}$ represent the weight matrices of the input spectra ( $X_{t}$ ), and the previous cell state ( $h_{t-1}$ ) passed to the gates $f_{t}$ , $i_{t}$ , $o_{t}$ , and $g_{t}$ . $b_{f}$ , $b_{i}$ , $b_{o}$ , and $b_{g}$ are the biases for each gate, σ  (sigmoid) and tanh are the nonlinear activation functions.

To evaluate the feasibility of the proposed SE-LSTM model, classical machine learning models (Partial least squares Discriminant Analysis, PLDA) and the unmodified LSTM were compared against it. Accuracy, loss value, and overfitting coefficient were adopted as evaluation criteria (Dhiman et al., 2023). To construct simplified models, characteristic wavelengths selection was performed using Successive Projections Algorithm (SPA), Genetic Algorithm (GA), Particle Swarm Optimization (PSO), and Bootstrapping Soft Shrinkage (BOSS) (Saha and Manickavasagan, 2021). SHAP, a game theory-based model interpretation framework, provides global and local interpretations by quantifying feature contributions to prediction outcomes (Salih et al., 2025). In this study, SHAP was employed to analyze the contribution of individual wavelengths to the model. All aforementioned algorithms were implemented in Python v3.8.20 with Scikit-learn v1.3.0 and PyTorch v2.2.0.

## 3 Results and discussion

### 3.1 Macro analysis based on VNIR-HSI

#### 3.1.1 Spectra analysis in VNIR band

**Figure 3:**
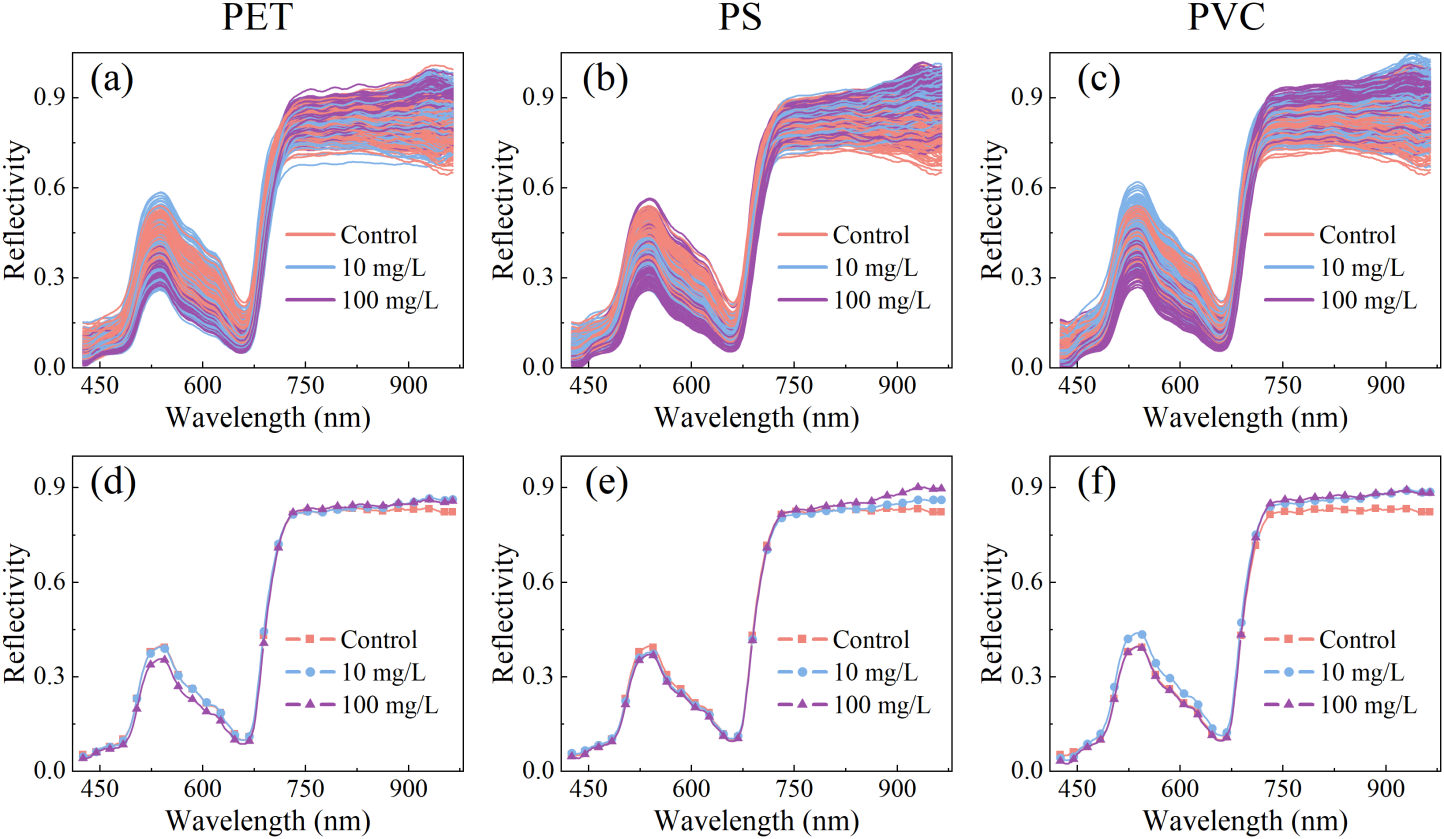
Original and mean spectra of leaves of rice seedling under PET **(a, d)**, PS **(b, e)**, PCV **(c, f)** MPs stress.

The leaf reflectance spectra of rice seedlings under normal growth conditions (control group) and exposed to microplastic (PET, PS, and PVC) stress at two concentrations (10 mg/L and 100 mg/L) are shown in **Figure 3**. The overall spectral trends of different treatment were similar and spectral peaks overlapped, which might be attributed to the dominant influence of leaf characteristics. In the blue-violet absorption region (425–490 nm), the peak of 480 nm is associated with the absorption of blue-violet light by leaves and the promotion of chlorophyll synthesis. Within the yellow-green absorption band (490–570 nm), the reflectance was observed to first increase and then decrease, with higher reflectance recorded in the green light region near 540 nm. This phenomenon was correlated with chlorophyll content in leaves and simultaneously explains the green coloration of rice leaves. The descending region at 530–660 nm, referred to as the “green edge”, was identified as a crucial indicator of chlorophyll absorption characteristics. The rapid reflectance increase in the 660–770 nm range, termed the “red edge”, was recognized as a reflection of carotenoid-related properties (Zolotukhina et al., 2024). In the near-infrared band (770–965 nm), reflectance characteristics were primarily influenced by the internal cellular structure of plants. Physiological and biochemical differences in rice leaves caused by different microplastic stresses were captured by visible-near infrared spectroscopy, establishing a technical foundation for microplastic stress detection.

#### 3.1.2 Dimensionality reduction clustering analysis

**Figure 4:**
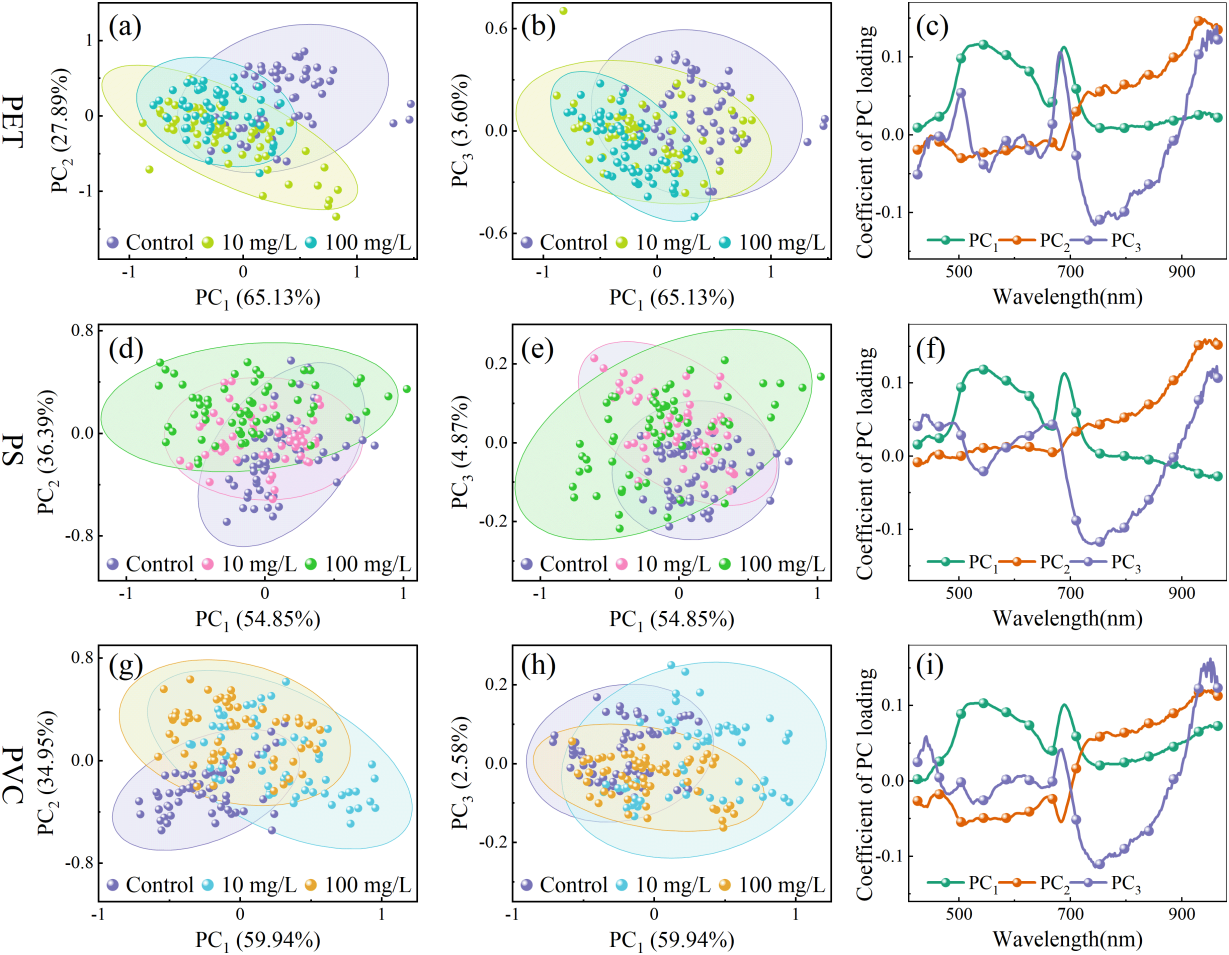
The scatter and loading plot of PET **(a–c)**, PS **(d–f)** and PVC **(g–i)** stress on rice seedlings.

To investigate the clustering distribution of rice leaves under MPs stress, PCA was performed to analyze the spectral characteristics under gradient concentrations of PET, PS, and PVC MPs. The dimensionality reduction results and corresponding loading curves are presented in **Figure 4**. The cumulative contribution rates of the first three principal components reached 96.62%, 96.11%, and 96.93% for PET, PS, and PVC respectively, accounting for the majority of the variances in the original spectral data. The substantial overlap observed under different microplastic stress levels may be attributed to the subtle spectral features induced by MPs. In the PC_1_ vs. PC_3_ plots of PET MPs, the clustering distribution exhibited a trend of shifting from the upper right to the lower left as the stress concentration increased. Meanwhile, in the PC_1_ vs. PC_2_ plots of PS MPs, a clockwise rotation pattern was showed with increasing concentrations. In contrast, a counterclockwise rotational trend was exhibited as stress levels increased in the PC_1_ vs. PC_2_ plots of PVC MPs.

The loading weights of the principal components reflect the relative importance of spectral features for clustering and indicate specific molecular and physiological changes in leaf tissues under microplastic stress. Under the three types of MPs stress, the loading curves exhibited generally consistent patterns. In the PC_1_ loadings, a trough at 660 nm corresponds to the strong absorption of chlorophyll a in the red spectral region (Zolotukhina et al., 2024). The increased reflectance may be related to chlorophyll degradation, potentially caused by MPs disrupting chloroplast membrane stability or inducing reactive oxygen species accumulation. The peak at 690 nm may reflect enhanced light scattering due to damage to the cell wall. The feature at 747 nm corresponds to the third overtone of the O–H bond, possibly indicating disruption of aquaporin formation in lipid membranes. In the PC_2_ loadings, the feature at 948 nm may arise from the third overtone of N–H bonds in proteins, suggesting that MPs stress may inhibit enzyme synthesis. In PC_3_, 505 nm is associated with absorption of carotenoids (Sytar et al., 2017), reflecting pigment changes induced by MPs. The features around 876 nm and 946 nm may be related to functional group interactions, such as the ester groups in PET or aromatic rings in PS, with polysaccharide O–H groups in plant cell walls. Although PCA provides a preliminary understanding of the clustering trends and associated spectral features, further methods development is needed to quantify stress levels more precisely.

### 3.2 MPs stress models development

#### 3.2.1 Full wavelength models

**Figure 5:**
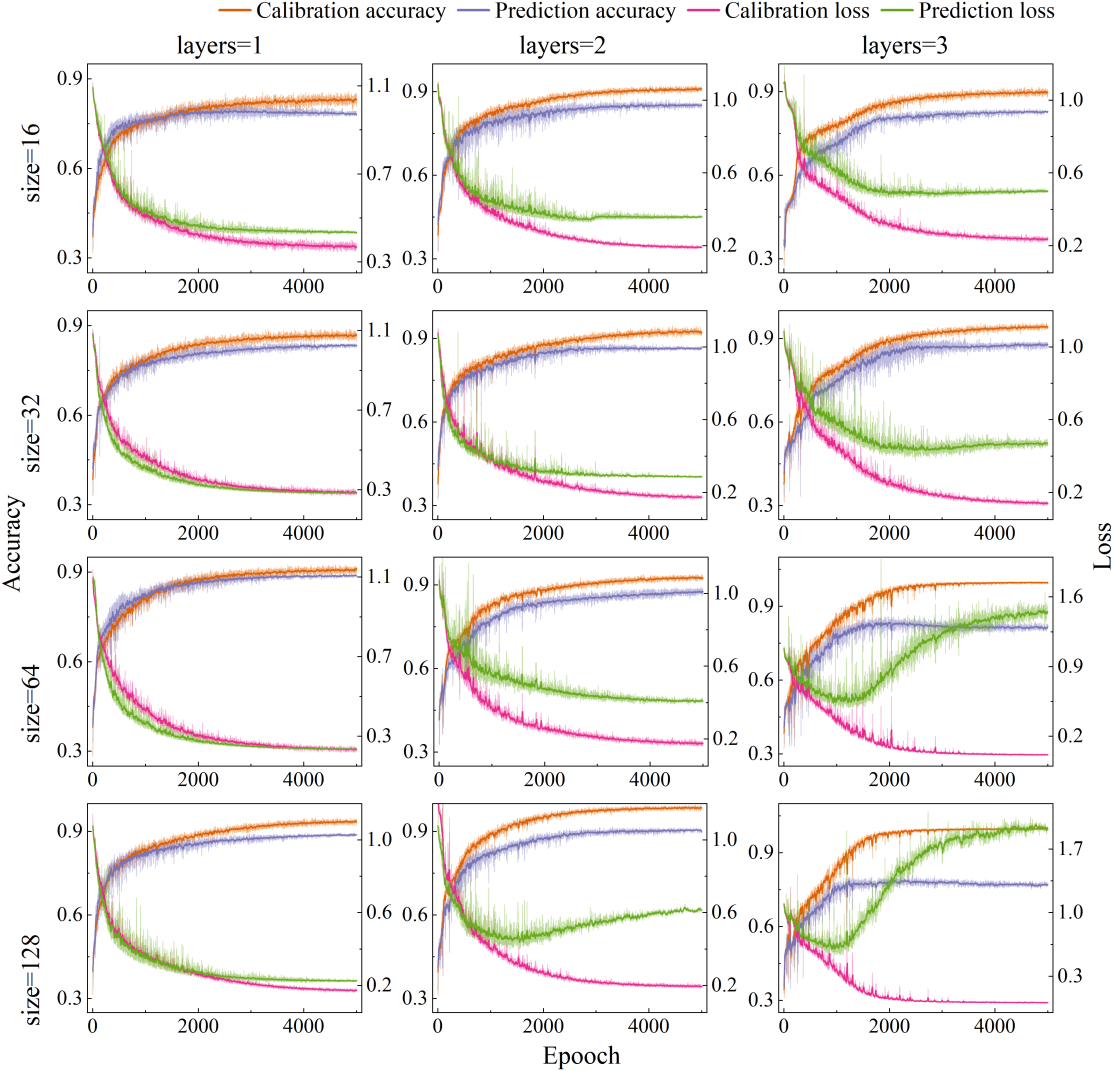
Accuracy and loss value of SE-LSTM model with different hidden layer and sizes.

The appropriate neural network structure and parameter have a significant impact on both detection performance and computational efficiency. There, the number of hidden layers and units per layer in the SE-LSTM model were systematically optimized. Based on empirical knowledge, the learning rate was set to 0.01 and the optimizer to Adam. PET MPs spectral data were as input, grid search algorithm was to explore different hidden layer configurations. The accuracy rates and loss values of the calibration set and prediction set under different hidden layer structures are shown in **Figure 5**, and the hidden layers 1, 2, and 3, as well as the number of hidden layer nodes 16, 32, 64, and 128, are discussed in detail. When the model had a single hidden layer, increasing the number of nodes led to gradual improvements in accuracy and reductions in loss, with stable trends indicating enhanced performance. For models with two hidden layers, accuracy improved with more nodes, peaking at 64 units, but further increases caused accuracy to decline and calibration - prediction set gaps to widen—signs of overfitting. A similar overfitting trend was presented in models with three hidden layers as the number of units increased. Overall, the configuration with two hidden layers and 32 units was optimal, achieving high accuracy, maintaining low loss value, and offering a good trade-off between performance and generalization.

**Figure 6:**
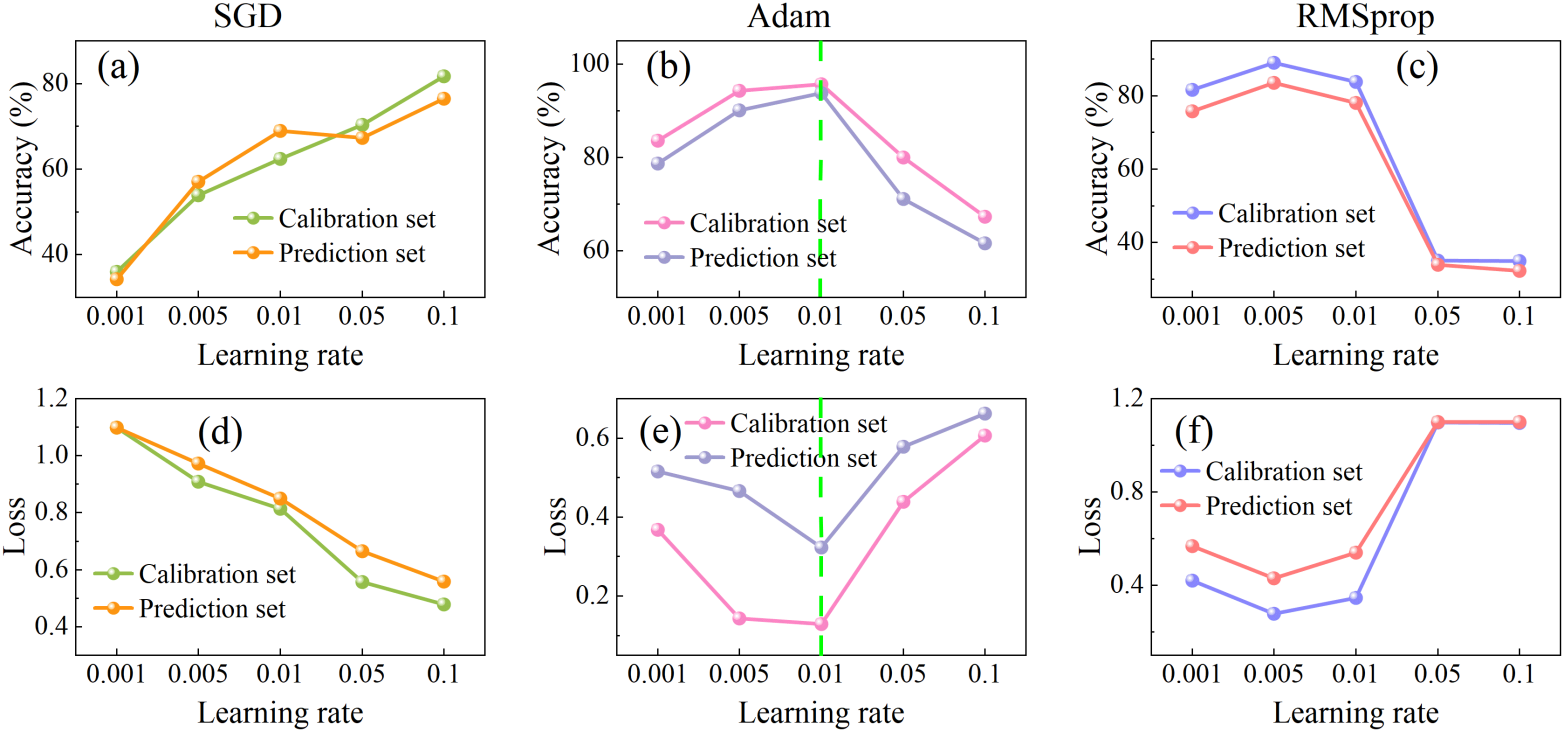
Accuracy **(a–c)** and loss value **(d–f)** of the SE-LSTM model with different learning rates (0.001, 0.005, 0.01, 0.05, 0.1) and optimizers (SGD: a,d; Adam: b,e; RMSprop: c,f).

Learning rates critically govern training stability and convergence dynamics, and loss functions fundamentally determine prediction-to-target fidelity. The compelling interdependence between these parameters necessitates dedicated research to strategically select optimizers and learning rates that maximize both convergence speed and final model accuracy. In this study, the performance of three commonly used optimizers (SGD, Adam, and RMSprop) was compared across five learning rates (0.001, 0.005, 0.01, 0.05, and 0.1), as illustrated in **Figure 6**. The performance of the SGD optimizer improved steadily with increasing learning rates, achieving a prediction set accuracy of 77.23% and the lowest loss of 0.55 at learning rate of 0.1. For the Adam optimizer, model accuracy initially increased and then decreased as the learning rate rose, while the loss value showed the opposite trend—decreasing first and then increasing. The optimal performance was reached at a learning rate of 0.01, where the prediction accuracy reached a maximum of 93.88% and the loss dropped to a minimum of 0.33. RMSprop exhibited a performance pattern similar to Adam but reached its peak at a learning rate of 0.005, with a validation accuracy of 84.5% and a loss of 0.53. Overall, the Adam optimizer combined with a learning rate of 0.01 demonstrated the best balance of accuracy and loss, which were selected for subsequent model training.

**Figure 7:**
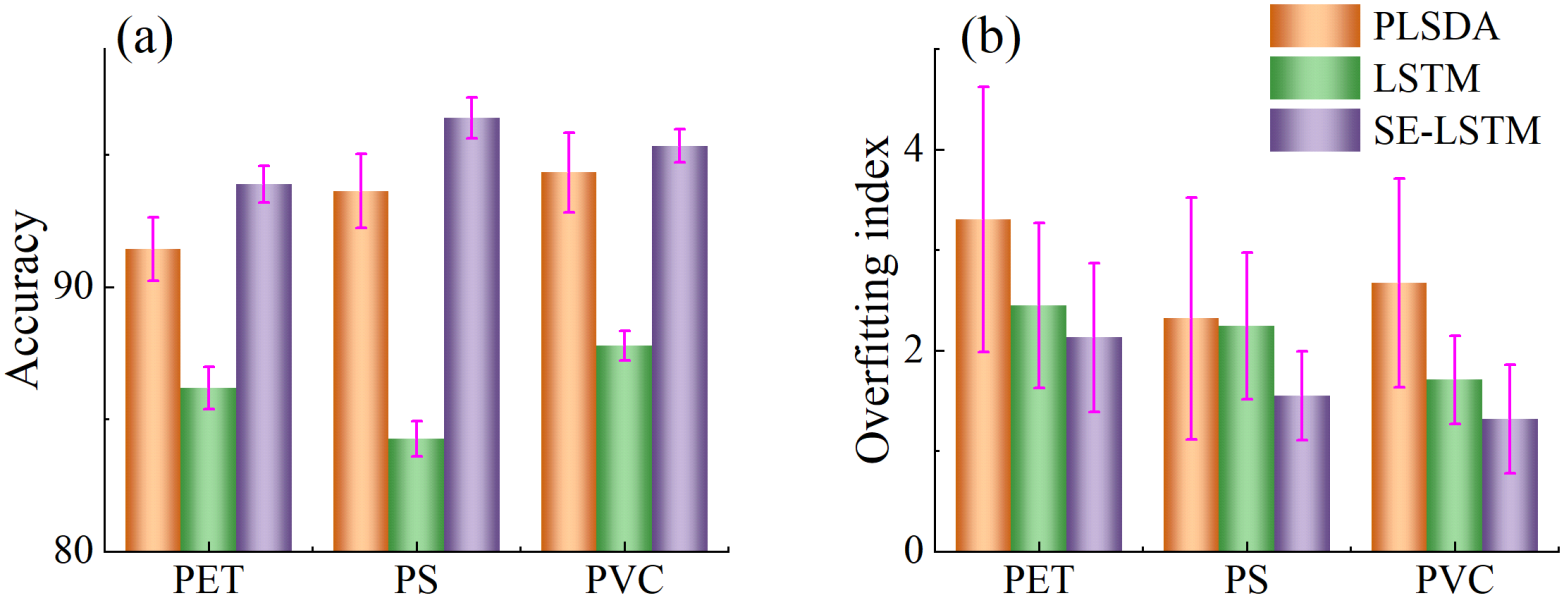
Accuracy **(a)** and overfitting index **(b)** of SE-LSTM model with different learning rates and loss function.

To comprehensively evaluate model performance, the accuracy and overfitting degree of three models on the prediction set were compared, as shown in **Figure 7**. The PLSDA model achieved accuracies of 91.43%, 93.88%, and 94.33% across the three datasets, with corresponding overfitting values of 3.30%, 2.32%, and 2.57%. Although the PLSDA model exhibited relatively high accuracy, it also holed the highest overfitting values, indicating weaker model stability. The LSTM model yielded accuracies of 86.19%, 84.27%, and 87.78%, with overfitting values of 2.45%, 2.37%, and 1.71%, respectively. While its accuracy was slightly lower than that of PLSDA, the lower overfitting values suggest better generalization capability. The SE-LSTM model achieved accuracies of 93.88%, 96.38%, and 95.33%, with the lowest overfitting values of 2.13%, 1.55%, and 1.32%, respectively. The SE-LSTM model demonstrated a balanced performance in both accuracy and stability, indicating superior capability in predicting MPs stress levels.

#### 3.2.2 Characteristic wavelengths and simplified models

**Figure 8:**
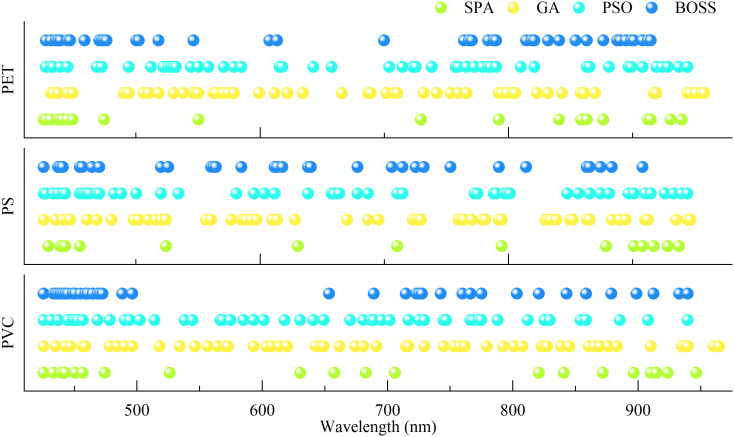
Selection of characteristic wavelengths for rice seedling stressed under PET, PS and PVC.

Although the predicting effect based on full wavelength has achieved satisfactory results, the large number of bands poses challenges for rapid detection. To enhance detection efficiency, characteristic wavelength selection was performed, and the feasibility of four algorithms, including successive projection algorithm (SPA), genetic algorithm (GA), particle swarm optimization (PSO), and bootstrapping soft shrinkage (BOSS), was evaluated. The characteristic wavelengths selected by different methods exhibited notable overlap (**Figure 8**). For example, under all three types of microplastic stress, wavelengths such as 425 nm, 431 nm, 439 nm, and 550 nm were frequently identified, suggesting that MPs exposure influences pigment absorption characteristics in rice leaves (Zhao et al., 2023), particularly those related to chlorophyll and carotenoids. Regarding differences among the wavelength selection methods, SPA tended to select a smaller number of bands, which likely emphasized on minimizing spectral redundancy and maximizing band independence. In contrast, a substantially larger number of wavelengths converging VNIR region were selected by GA and PSO, which is likely due to their global search strategies for the identification of stress-related features. The BOSS algorithm showed a degree of stochasticity in band selection; however, the wavelengths selected overlapped with those from others. This suggests that BOSS balances the retention of critical spectral information with greater selection flexibility.

**Figure 9:**
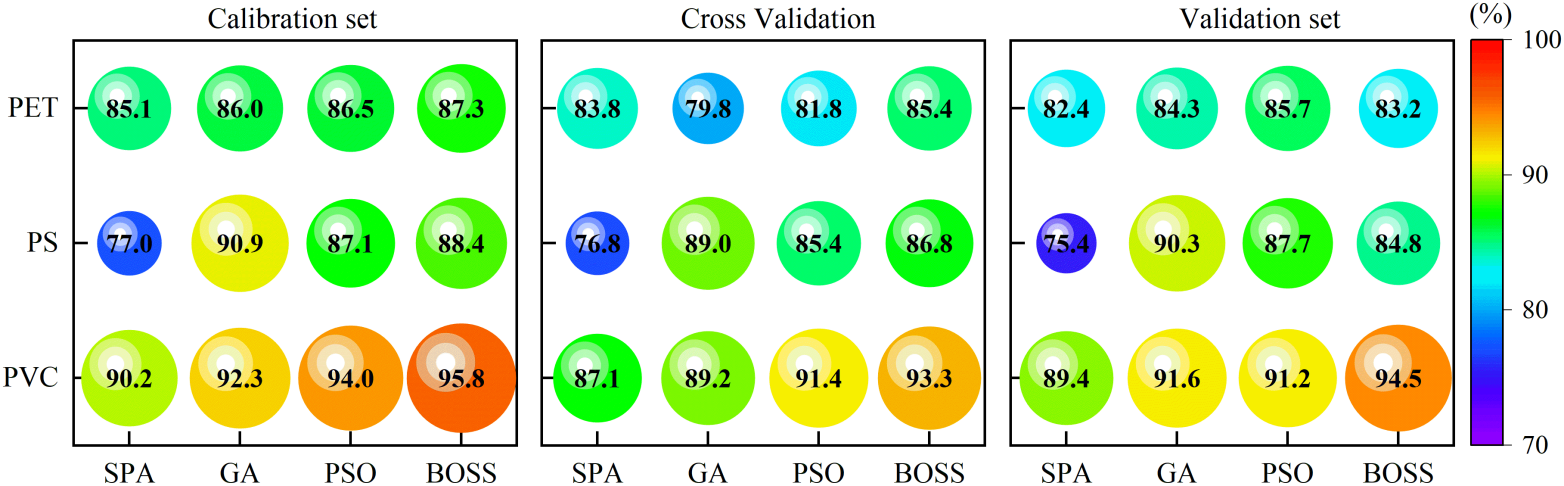
Performance for the classification models based on characteristic wavelengths.

The accuracy of the calibration set, cross-validation set and validation set for stress concentration prediction with characteristic wavelengths were shown in **Figure 9**. Compared to full-wavelength models, the multi-wavelength model significantly reduces the number of inputs and maintains a relatively high classification accuracy rate. The optimal prediction accuracies for PET, PS, and PVC were 85.7%, 90.3%, and 94.5% in the multi-wavelength detection model, respectively. Regarding the unsatisfactory prediction of PET, the accuracy rate of full-wavelength model is 93.88%, while that of multi-wavelength model is only 83.15%. In contrast, PVC prediction by two models almost achieved the same prediction results, with full-wavelength and multi-wavelength models achieving 95.33% and 94.51%, which was almost comparable. Among the wavelength selection methods, the GA achieved the optimal results for PET and PS, whereas the BOSS algorithm performed optimally for PVC, which can be used for subsequent analysis. These findings suggest that multi-wavelength models significantly reduced data dimensionality and preserving critical spectral information, enabling efficient and rapid detection.

#### 3.2.3 Models explanation

**Figure 10:**
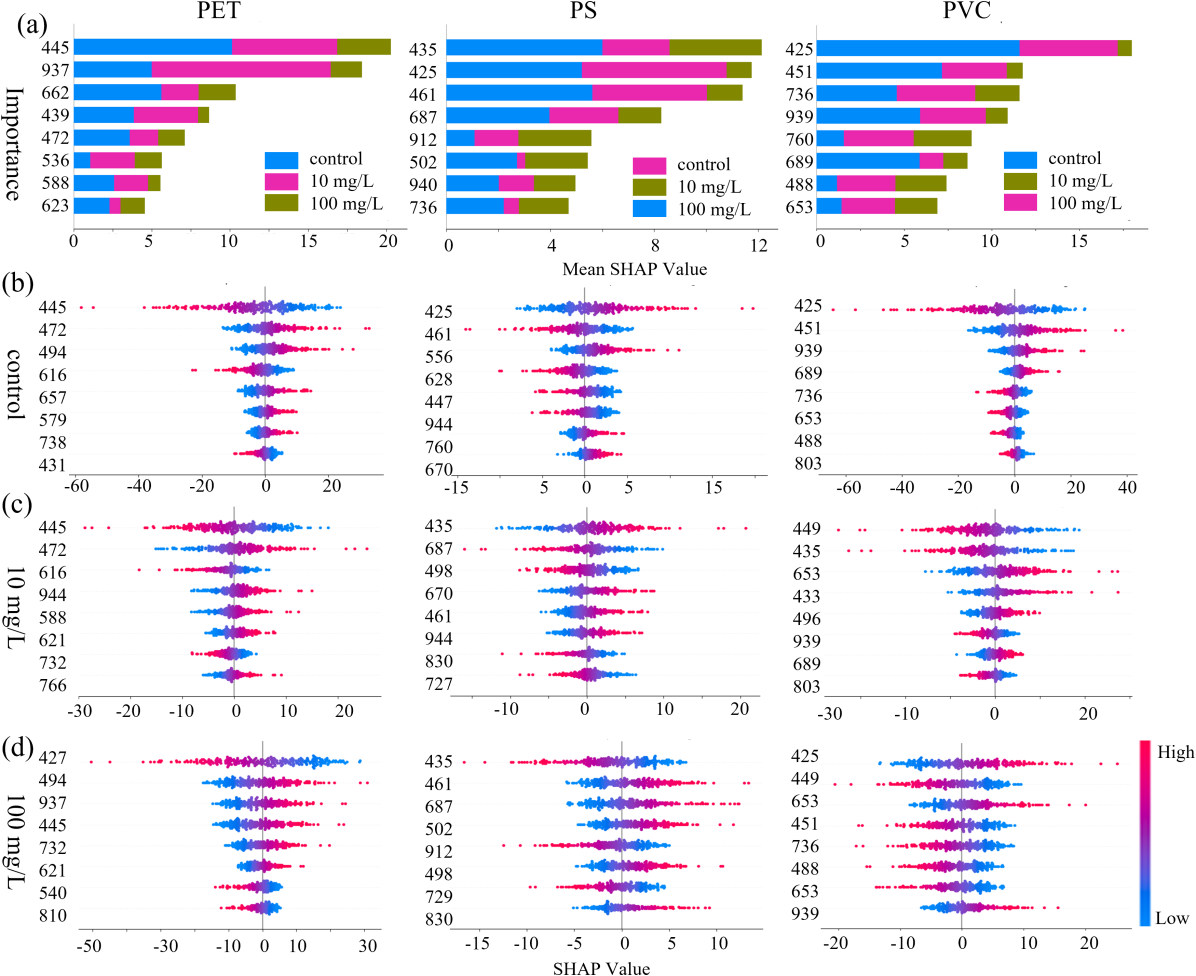
Importance distribution of characteristic wavelength based on SHAP values for **(a)** the overall dataset, **(b)** the control group, **(c)** the 10 mg/L group, and **(d)** the 100 mg/L group.

To explore the importance of characteristic wavelengths and their corresponding biochemical associations under MPs stress, the SHAP analysis was employed to enhance model interpretability. As shown in **Figure 10**, characteristic wavelengths contributing to stress detection in the spectral data were identified and their potential associated components were analyzed. The global importance of individual wavelengths was visualized in the bar chart (**Figure 10a**), and **Figures 10b–d** illustrates feature importance variations across stress levels (0, 10, and 100 mg/L) using bee swarm plots. From a global perspective, the top eight discriminative wavelengths for PET, PS, and PVC are as follows: PET: 445, 472, 536, 588, 623, 662, and 937 nm; PS: 425, 435, 461, 502, 687, 736, 912, and 940 nm; PVC: 425, 451, 488, 653, 689, 736, 760, and 939 nm. Notably, there is considerable overlap among the three groups in the 425–502 nm (blue light) range, potentially associated with chlorophyll a/b and carotenoid absorption; in the 687–760 nm (red-edge) region, likely related to chlorophyll fluorescence sensitivity; and in the 736–940 nm (near-infrared) region, which may correspond to water or cellulose overtone vibrations (He et al., 2024). These findings suggest that stress-induced disruptions in pigment metabolism, cellular structure, and water transport may serve as effective indicators for distinguishing stress levels. In terms of differences, PET detection showed unique contributions at 445 nm (a chlorophyll absorption peak in the blue-violet region) and 937 nm (a third overtone of O–H bond vibrations). For PS, a strong contribution was observed at 912 nm, corresponding to C–H stretching vibrations. Additionally, the feature at 760 nm exhibited heightened sensitivity for PVC, which attributed to the second overtone of O–H vibrations, indicating possible shifts in dominant physiological responses across different stress concentrations.

The bee swarm plots further revealed the concentration dependence of feature contributions: from top to bottom is the distribution of wavelength importance, and each point in the map represents the SHAP value of the sample at each wavelength. The distribution of SHAP values reflects the contribution level through color mapping, with red representing high values and blue representing low values. The positions of the positive and negative half-axes respectively represent the promoting or inhibitory effects of the features on the model prediction. In PET detection, the distribution of SHAP values in the carotenoid absorption valley at 472 nm and the flavonoid sensitive zone at 439 nm in the control group showed high synchrony, which might be related to the homeostasis of secondary metabolites. The strong negative contribution of the chlorophyll reflection peak at 540nm and the water stress marker at 937nm in the 100 mg/L group suggests that high concentrations of microplastics may intensify the stress response by damaging photosynthetic organs and intensifying transpiration. Meanwhile, there are differences in the single weight distribution of different types and concentrations of MPs. This may be related to the dynamic differentiation of the SHAP contribution mode and the nonlinear weight distribution of the model for capturing physiological markers at different stress stages.

### 3.3 Microscopic analysis base on SR-FTIR

The biochemical response characteristics such as photosynthetic inhibition, osmotic stress and cellular structure damage of leaves caused by MPs stress had been explored in VNIR bands. However, limited by the characteristics of the molecular vibrational overtone region of the VNIR spectra, it can only capture the weak absorption signals of bonds such as C-H, O-H and N-H, making it difficult to accurately detect the interaction mechanism of MPs particles on plant tissues. The introduction of SR-FTIR can break through the limitation of spatial resolution. Taking PET MPs stress as a typical representative, it can achieve the selective enrichment law in the vascular bundles of leaf veins and the thin-walled cells of mesophyll through high-sensitivity micro-area imaging, as well as the characterization of molecular interaction interfaces and the verification of metabolic disturbance excitation.

#### 3.3.1 One dimensional spectral analysis

**Figure 11:**
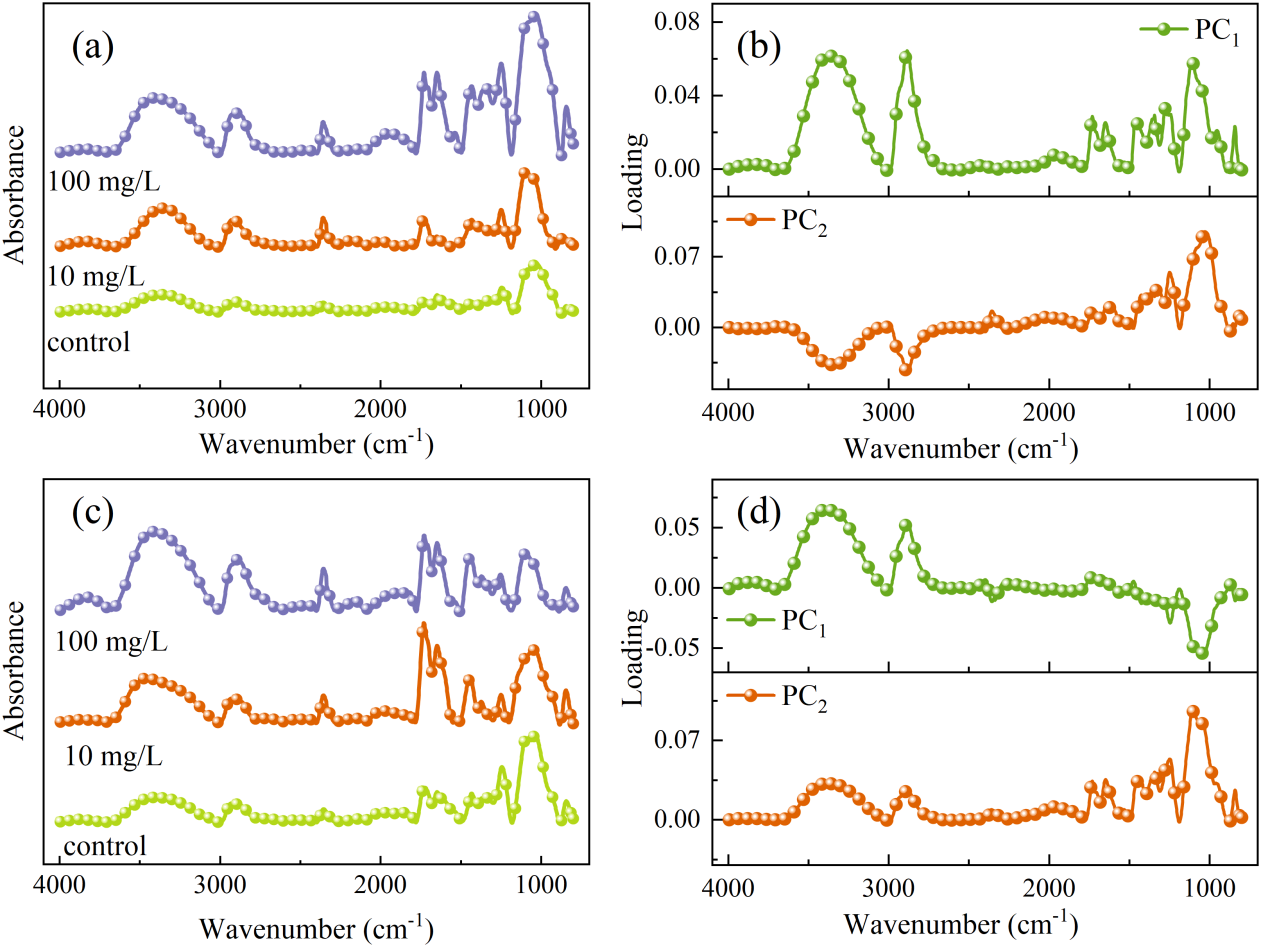
One dimensional average spectra and PCA loading plots of leaf veins **(a, b)** and mesophyll **(c, d)**.

The overall trends of the mid-infrared spectra in the vein and mesophyll regions of rice leaves under PET microplastic stress were similar (**Figure 11**), but different spectral trends were presented in the functional group region (4000–2800 cm^-1^) and the fingerprint region (1800–600 cm^-1^). The absorption peak intensities of the veins and mesophyll in the control group were generally low. However, multiple characteristic peaks showed with PET concentration increased, indicating that MPs triggered dynamic responses of the molecular structure at the subcellular scale. In the vein spectra (**Figure 11a**), the peak intensity of the O-H/N-H stretching vibration at 3352 cm^-1^ significantly increased with concentration increased, which might be related to the obstruction of water transport in the vascular bundle or the accumulation of woody fluid (Bunaciu et al., 2012). Combined with the PCA loading (**Figure 11b**), the synergistic enhancement of the peak at 1776 cm^-1^ in PC_1_ loading of leaf vein and the stretching vibration of C=O of the ester group at 1730 cm^-1^ in original spectra points to the physical adsorption and chemical hydrolysis process of the PET bulk or its degradation products on the inner wall of the fiber tube (Mecozzi and Nisini, 2019). Furthermore, the stretching vibration of C-O at 1248cm^-1^ in PC_2_ loading and the synchronous increase of the 1244 cm^-1^ peak in the original spectra indicate that PET induces enhanced lignification of the secondary wall of vascular bundle sheath cells through mechanical friction.

The spectral response in the mesophyll region (**Figure 11c**) was dominated by membrane system damage and dynamic regulation of the cell wall. The asymmetric stretching vibration of CH_2_ of lipid at 2898 cm^-1^ reflects the accumulation of malonaldehyde in chloroplasts (Brandl, 2013), which is consistent with chlorophyll scattering at 690 nm in VNIR spectra. The C-H bending vibration of pectin methylation at 1469 cm^-1^ in PC_1_ loading suggests that a decrease in cell wall rigidity to alleviate PET induced stress. The stretching vibration of C=O of the ester group at 1730 cm^-1^ and the bending vibration of C-H of the benzene ring at 841 cm^-1^ are related to PET. The peak intensity increases with the increase of concentration in the vein, while only weakly appears or is completely absent in the mesophyll. In conclusion, PET MPs are more likely to be transported through the vascular bundle system and enriched on the inner walls of the vessels, while there are fewer mesophyll cells, which may be related to the rejection of large-sized particles by exocytosis.

#### 3.3.2 Two dimensional spectral analysis

Due to the overlapping of multiple absorption peaks in SR-FTIR spectra and the unclear mechanism of biochemical changes in leaves. The 2DCOS technology was used to explore the biochemical change mechanism of leaf veins and leaves caused by external disturbances of rice under different concentrations of MPs. In addition, the absorption peaks in the functional group region (4000–2800 cm^-1^) and the fingerprint region (1800–800 cm^-1^) are of different complexities, so, they are explored in two regions. The automatic peak in the synchronous spectra represents the spectral intensity at the corresponding coordinate, while the asynchronous spectra represent the sequence of component peak intensity changes caused by external disturbances.

**Figure 12:**
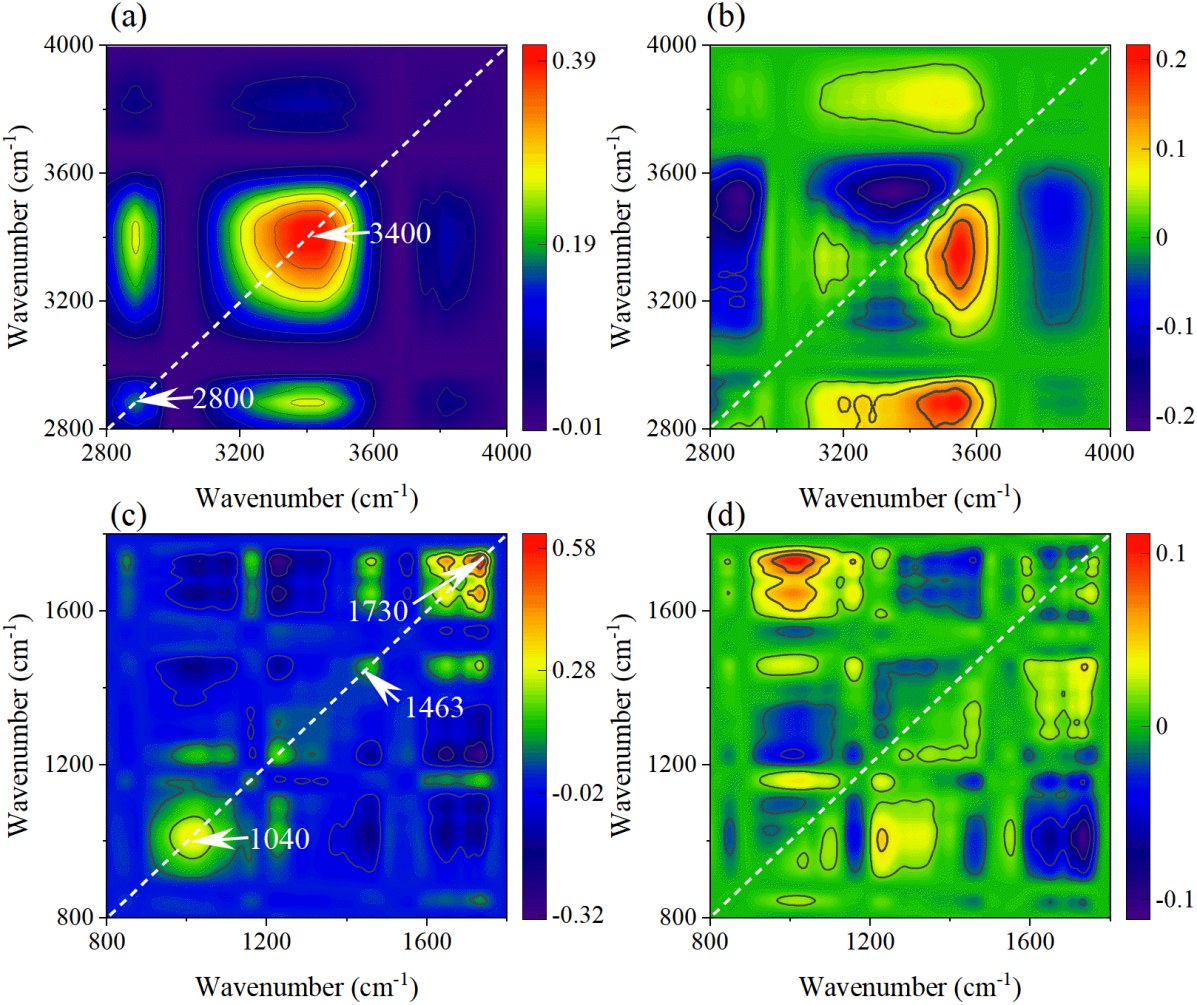
The synchronous **(a, c)** and asynchronous **(b, d)** 2DCOS maps generated from the SR-FTIR spectra of leaf veins in the functional group region and fingerprint region.

The 2DCOS spectra of the functional group regions of leaf veins under PET MPs stress were shown in **Figures 12a, b**. The two automatic peaks of 3433 cm^-1^ and 2918 cm^-1^ in the synchronous spectra correspond respectively to the stretching vibration of O-H/N-H and the asymmetric stretching vibration of CH_2_, indicating that the change of water state and the disorder of membrane lipid metabolism are the response characteristics of leaf veins. The relationship of component changes was clarified from 3356 → 2910 → 2863→ 3169 → 3506 cm^-1^ in the asynchronous spectra. The O-H vibration at 3356 cm^-1^ reflected the interference of PET MPs on the water transport of vascular bundles. Subsequently, CH_2_ at 2910 cm^-1^ and 2863 cm^-1^ marked the initiation of membrane lipid peroxidation, and N-H vibration at 3169 cm^-1^ indicates the conformational change of membrane-binding proteins. The O-H vibration at 3506 cm^-1^ may reflect the increase in the hydroxylation degree of polysaccharides in the cell wall, which is the adaptive response of vascular bundle tissues to persistent stress.

In the synchronous spectra of the leaf vein fingerprint region (**Figure 12c**), there are automatic peaks such as C-H curvature of the benzene ring of PET at 840 cm^-1^, the C-O of lignin at 1258cm^-1^, and the C=O of ester group of PET at 1724 cm^-1^. The accumulation of MPs in the vascular bundle and the defense responses it triggers are demonstrated. The asynchronous spectra (**Figure 12d**) revealed the dynamic change processes of the cell wall components and metabolites (1115→1439→1373→1252→1648 →975 cm^-1^). Firstly, the cellulose vibration at 1115 cm^-1^ indicates the remodeling of polysaccharides in the cell wall. Subsequently, the methylation of pectin at 1439 cm^-1^ further regulates the performance of the cell wall. Then, the lignin-related vibrations at 1252 cm^-1^ revealed the strengthening process of the secondary cell wall. Finally, the protein denaturation at 1648 cm^-1^ and the PET benzene ring characteristics at 975 cm^-1^ suggested the enzyme function impairment and the accumulation of MPs degradation products caused by continuous stress.

**Figure 13:**
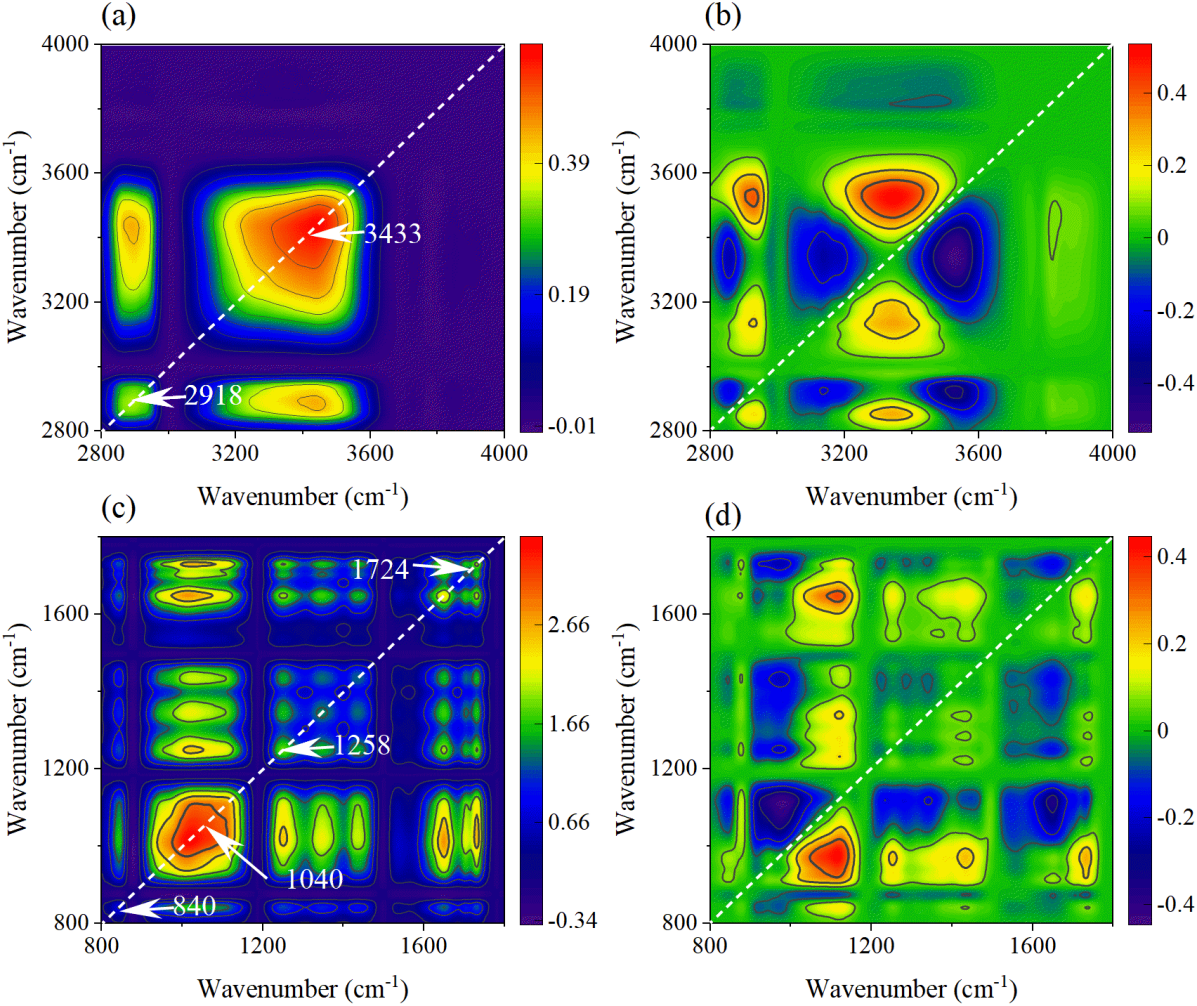
The synchronous **(a, c)** and asynchronous **(b, d)** 2DCOS maps generated from the SR-FTIR spectra of leaf mesophyll in the functional group region and fingerprint region.

The responses of mesophyll tissue in the functional group regions (**Figures 13a, b**) differ from those in the veins. The automatic peaks of O-H stretching at 3400 cm^-1^ and CH_2_ stretching at 2880 cm^-1^ in the synchronous spectrum indicate that the PET stress of mesophyll cells is mainly concentrated on the cell wall and membrane system. The sequence of peak changes in the asynchronous spectrum (3443→3187→3359→2887→3839 cm^-1^) reveals the response path: Firstly, it causes the rapid adjustment of free hydroxyl groups in the polysaccharide at 3443 cm^-1^. Secondly, it leads to the vibration of N-H of protein at 3187 cm^-1^, indicating that MPs causes cell wall damage in mesophyll tissue. Then, the vibration of membrane lipids at 2887cm^-1^ and hydroxyl groups at 3839 cm^-1^ led to the damage of mesophyll cells under continuous stress, which contrasted with the vein tissue.

In the synchronous spectra (**Figure 13c**) of the mesophyll fingerprint region, the auto-peaks such as C-O-C of cellulose at 1040 cm^-1^, CH_2_ bending of pectin at 1463 cm^-1^, and C=O of ester group at 1730 cm^-1^ were associated with concentration dependence, reflecting the effect of PET stress on the mesophyll cell wall. The sequence of component changes in the asynchronous spectra (**Figure 13d**) is 1226→1087→1020→968→1158→1337→1738→1647→1463 cm^-1^. Firstly, the vibrations of lignin/phenolic acid at 1226 cm^-1^ and the cellulose at 1087 cm^-1^ indicate that secondary metabolic activation and cell wall enhancement are the initial defense strategies. Then, 1020 cm^-1^ and 968 cm^-1^ suggested the remodeling of the carbon metabolism pathway, which might be a response to insufficient energy supply. Finally, the vibration of ester group at 1738 cm^-1^ and the protein denaturation at 1647 cm^-1^ revealed the cumulative damage of PET chemical toxicity to mesophyll cells.

## 4 Conclusions

In this study, an interpretable and rapid method for detecting rice seedlings under MPs stress was proposed by integrating macro-scale VNIR-HSI, micro-scale SR-FTIR and deep learning. The specific physiological and biochemical reactions of rice leaves to PET, PS and PVC MPs were revealed by VNIR-HSI. The subtle spectral changes of rice seedling leaves caused by MPs stress were explored by PCA, and chlorophyll degradation, pigment absorption changes and water transport interference were reflected in the loading curve. An improved SE-LSTM full-spectral model was developed to achieve rapid detection, with an accuracy rate of >93.88%. By extracting characteristic wavelengths using four feature selection algorithms, a simplified model was established to reduce the input dimension and maintaining robust performance. The interpretability framework of SHAP highlights the characteristic wavelengths related to chlorophyll, carotenoids, water and cellulose, and reveals the dynamic physiological damage under different stress levels. At the microstructure level, the combination of SR-FTIR spectroscopy and 2DCOS provides detailed insights into the molecular mechanism by MPs stress. The different stress responses of components in the mesophyll and vein tissues of leaves at the subcellular level have been revealed. In conclusion, the combination of VNIR-HSI, SR-FTIR and deep learning provides a feasible solution for the early detection of MPs stress in crops. This work has laid a technical foundation for the diagnosis of other plant stresses and provided a direction for the detection of emerging pollutants in agricultural sustainability and food safety.

## Data Availability

The original contributions presented in the study are included in the article/supplementary material. Further inquiries can be directed to the corresponding authors.
